# Targeting inflammatory macrophages with hyaluronan tetrasaccharide: effects on fibroblast collagen degradation and synthesis

**DOI:** 10.3389/fimmu.2025.1592751

**Published:** 2025-06-05

**Authors:** Eiko Uno, Florence Kim, Mihoko Yoshino, Yasunari Sato, Masao Hashimoto, Kenji Watanabe, Yoichi Mizukami, Jun Muto

**Affiliations:** ^1^ Fundamental Technology Research Division, ROHTO Pharmaceutical Co., Ltd., Kizugawa, Kyoto, Japan; ^2^ Institute of Gene Research, Yamaguchi University Science Research Center, Yamaguchi, Japan; ^3^ Department of Dermatology, Ehime University Graduate School of Medicine, Toon, Ehime, Japan

**Keywords:** hyaluronan tetrasaccharide (HA4), macrophages, fibroblasts, dermis, photoaging

## Abstract

Hyaluronan (HA) provides moisturizing benefits and exhibits unique biological activities based on its molecular weight. While the anti-inflammatory effects of high-molecular-weight HA have been well studied, the impact of hyaluronan tetrasaccharide (HA4), an ultralow-molecular-weight HA, on the skin immune system is not fully understood. Thus, we investigated how HA4 affects the differentiation of M1 macrophages, which increase during photoaging. As a result, we added HA4 during the M1 macrophage differentiation phase and conducted a gene expression analysis. HA4 partially decreased the transition from M0 to M1 macrophages and reduced the expression of proinflammatory cytokines like IL-6. However, the M2 marker IL-1ra increased, while IL-10 levels remained constant, suggesting that HA4 does not fully polarize macrophages toward the M2 phenotype. Normal human dermal fibroblasts (NHDF) were treated with an M1 macrophage-conditioned medium (M1-CM) and a modified version containing HA4 (M1+HA4-CM). The M1+HA4-CM notably decreased the expression of IL-6 and IL-8, along with the collagen-degrading enzyme MMP1. Collagen synthesis assays showed that HA4 helped restore collagen fiber formation. Moreover, RNA-seq analysis of NHDF treated with the conditioned medium confirmed that M1+HA4-CM amplified the expression of genes related to collagen production while decreasing collagen-degrading enzyme gene expression. Neutralization assays employing a TLR4 antibody suggested that decreasing IL-6 in NHDF by HA4 may be independent of the TLR4 signaling pathway. HA4 is vital in partially suppressing M1 macrophage differentiation and the release of inflammatory factors, as well as regulating collagen remodeling in NHDF. These findings indicate that HA4 holds promise as a molecule for mitigating inflammation-induced collagen degradation by modulating macrophage activity in photoaged skin.

## 1 Introduction

Hyaluronan (HA) is a long, linear polysaccharide made up of repeating disaccharide units of D-glucuronic acid and N-acetyl-D-glucosamine, with a molecular weight that can reach as high as 2 × 10^7^ Da (1, 2). HA is a key element of the extracellular matrix (ECM) in skin tissue and is produced by hyaluronan synthases (HAS) found in the inner membranes of keratinocytes and fibroblasts (1). Thanks to its remarkable water retention ability, HA is commonly included in moisturizers and serums, boosting skin hydration and improving elasticity.

In addition to its moisturizing effects, HA has been extensively researched as a bioactive substance, with its effectiveness significantly influenced by its molecular weight and interactions with receptors (1). HA regulates physiological responses associated with immune function and tissue inflammation (1). For example, prior research has demonstrated that high-molecular-weight HA (100–1,000 kDa) inhibits UVB-induced DAMP-dependent inflammation in keratinocytes through the CD44v pathway (3). Conversely, low-molecular-weight hyaluronic acid (10 kDa–100 kDa) demonstrates proinflammatory effects, facilitating angiogenesis and tissue remodeling in wound healing (4). Among these, 6-mer oligo-HA is known to function as an inflammation-inducing factor in human chondrocytes via Toll-like receptor 4 (TLR4) and CD44 (5). Moreover, small HA oligomers (sHA; 4–16 saccharide units) produced in inflamed tissues have been demonstrated to promote the maturation of dendritic cells (DC) without relying on CD44 or RHAMM receptors (1, 6).

Conversely, studies indicate that ultralow-molecular-weight HA (HA4) does not trigger inflammatory signaling in keratinocytes but shows anti-inflammatory effects through TLR4 (3). These findings suggest that HA’s biological activities are mediated by various receptors, which depend on its molecular weight and the cell type.

Recent research emphasizes the vital function of macrophages, the predominant immune cell type found in human dermis, in skin photoaging. Photoaged skin displays an imbalance between two macrophage types: M1, linked to proinflammatory reactions, and M2, recognized for their anti-inflammatory effects (7). Notably, there is a noted rise in M1 macrophages and a decline in M2 macrophages. This imbalance in macrophage populations is believed to harm collagen metabolism, impacting its production, breakdown, and elimination, which may ultimately lead to photoaging signs like diminished skin elasticity.

Specifically, the higher percentage of M1 macrophages in the photoaged dermis is believed to maintain chronic inflammation, triggering inflammatory responses in nearby cells, such as fibroblasts (8). Nevertheless, the impact of reducing M1 macrophage-induced inflammation on fibroblasts is still largely unexamined. Additionally, although earlier research has shown that HA exhibits varied biological activities based on its molecular weight, the effect of HA4 on the skin’s immune system is still unclear.

This study sought to clarify HA4’s potential influence on macrophage differentiation, especially its suppression of the differentiation of M1 macrophages that expedite photoaging. Additionally, it explored how HA4 affects fibroblast inflammation and collagen remodeling triggered by substances released from M1 macrophages.

## 2 Material and methods

### 2.1 Cell culture

Normal Human Dermal Fibroblasts (NHDF) were sourced from KURABO in Osaka, Japan. These NHDF were cultured in Dulbecco’s modified Eagle’s medium enriched with 10% fetal bovine serum (FBS), penicillin (100 IU/mL), and streptomycin (100 μg/mL) and incubated at 37°C with 5% CO_2_. The human acute monocytic leukemia cell line, THP-1, was obtained from the American Type Culture Collection (Manassas, VA, USA). THP-1 cells were maintained in RPMI-1640 medium fortified with 10% FBS, penicillin (100 IU/mL), streptomycin (100 μg/mL), and β-mercaptoethanol (50μM), and they were also grown in an incubator with 5% CO_2_ at 37°C.

### 2.2 Reagents

ROHTO Pharmaceutical Co., Ltd. synthesized and purified HA4 (0.8 kDa), a LPS-free tetrasaccharide.

### 2.3 Macrophage differentiation

**Figure 1:**
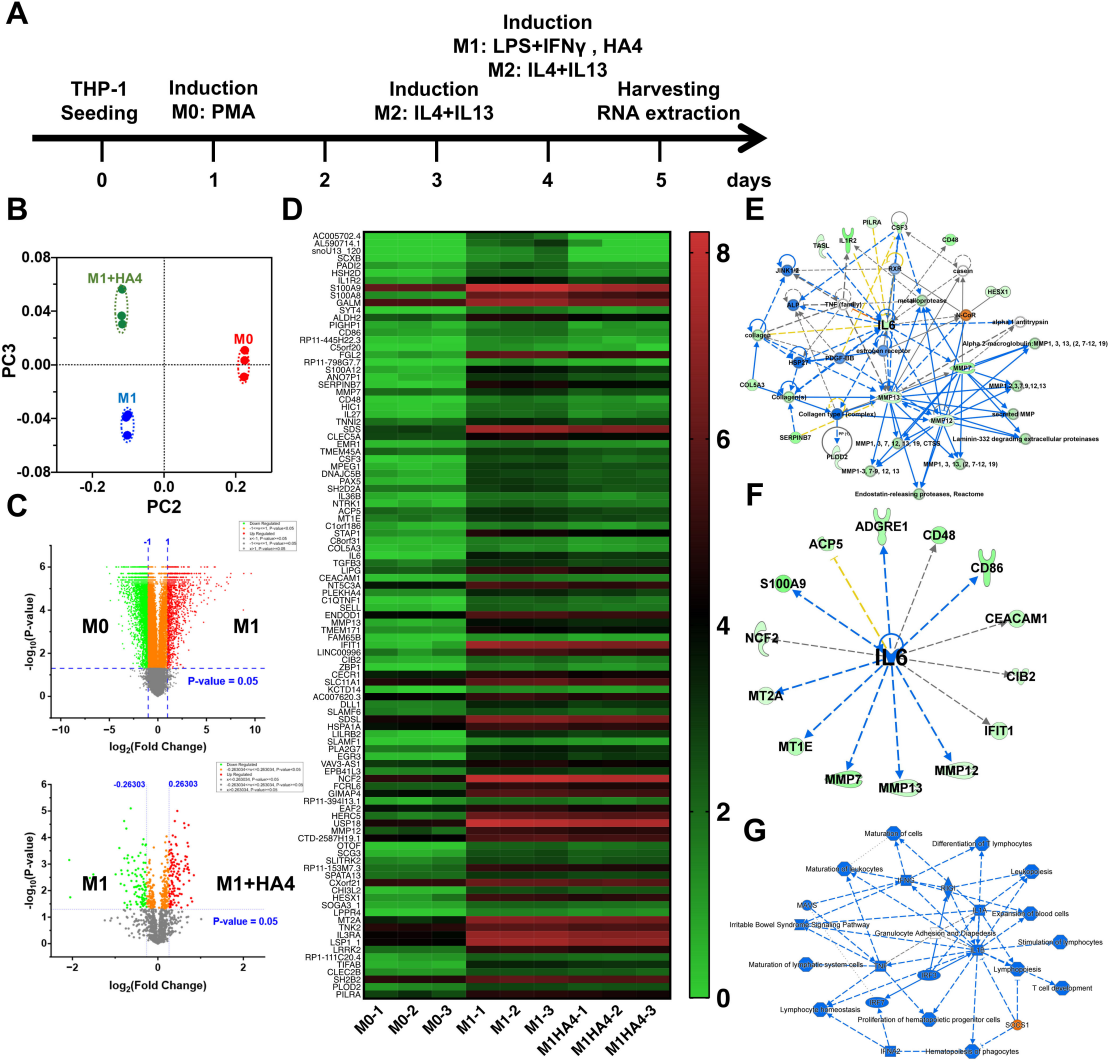
Impact of hyaluronan on macrophage polarization and cytokine expression. **(A)** Schematic overview of the experimental setup for differentiating macrophages from M0 to M1/M2 phenotypes using hyaluronan (HA) treatment. **(B)** PCA analysis of RNA-seq data illustrating gene expression profiles in M0, M1, and M1 macrophages treated with hyaluronan tetrasaccharide (HA4). **(C)** Volcano plot illustrating the comparison of gene expression between M0 and M1 or M1 and M1+HA4 macrophages. **(D)** Heatmap showing gene expression variations between M0, M1, and M1+HA4 macrophages. **(E, F)** Network analysis or **(F)** upstream analysis emphasizing the role of IL-6 in M1 macrophages treated with HA4. **(G)** Detailed graphical summary of transcriptional changes and relevant biological pathways in M1 macrophages treated with HA4.

THP-1 cells were differentiated into M0 macrophages by treating them with 5 ng/mL phorbol 12-myristate 13-acetate (PMA) for 72 hours. To achieve M2 polarization, M0 macrophages were stimulated twice with 25 ng/mL interleukin-4 (IL-4) and 25 ng/mL interleukin-13 (IL-13), first at 48 hours and again at 72 hours post-initial differentiation. For M1 polarization, M0 macrophages received treatment with 10 ng/mL lipopolysaccharide (LPS) and 20 ng/mL interferon-γ (IFN-γ) for 24 hours. The impact of HA4 was evaluated during M1 polarization, conducted with and without 0.1% (w/v) or 1% (w/v) HA4 added for 24 h at initiation. RNA extraction was performed 24 hours after the last stimulation for M1 and M2 group macrophages. The experimental scheme of macrophage differentiation is shown in **Figure 1A**.

### 2.4 Coculture with CM

Before use, M1-CMs cultured with or without HA4 for NHDF coculture were diluted in a fibroblast culture medium. The CM was diluted 1:64 for RNA-seq, real-time qPCR analysis, ELISA, and neutralizing antibody assays. For other experiments, a 1:16 dilution was used.

### 2.5 ELISA kits

We assessed the concentrations of IL-6, IL-8, TNFα, IL-1ra, and MMP1 in the conditioned medium using ELISA kits from R&D Systems in Minneapolis, MN, USA. (IL-6, D6050; IL-8, DY208; IL-1ra, DRA00B; TNFα, DTA00D; MMP1, DY901B-05), following the manufacturer’s instructions. In summary, samples and standards were introduced to microplate wells coated with antibodies and allowed to incubate. A detection antibody was introduced following a washing step, resulting in another incubation and wash. A substrate solution was applied, and the reaction was halted after the advised duration. Finally, absorbance was recorded at the designated wavelength using a microplate reader.

### 2.6 Western blot analysis

Total protein was extracted from NHDF using an M-PER protein extraction reagent (Thermo Fisher Scientific, 78503, Waltham, MA, USA) combined with protease and phosphatase inhibitors. Western blot analysis was conducted with the WES system (ProteinSimple, San Jose, CA, USA) per the manufacturer’s guidelines. In summary, protein samples were prepared in a sample buffer, denatured, and loaded into the WES capillary system. The separation, immunoprobing, and detection processes were performed automatically using the designated primary and secondary antibodies. Data analyses were conducted using Compass software (ProteinSimple, San Jose, CA, USA), utilizing αTubulin as a loading control. The following antibodies were employed: anti-IL-6 antibody (AF-206-SP), anti-TRAF6 antibody (8028S), and anti-TNFAIP3/A20 antibody (5630) (all from Cell Signaling Technology, Danvers, MA, USA); and anti-αTubulin antibody (Abcam, ab7291, Cambridge, UK).

### 2.7 Real-time qPCR analysis

Total RNA was extracted from macrophages and NHDF with the Maxwell^®^ RSC Instrument (Promega, Madison, WI, USA) following the manufacturer’s guidelines. The complementary DNA (cDNA) library was prepared using ReverTra Ace^®^ qPCR RT Master Mix (Toyobo, Osaka, Japan) from 100–500 ng of total RNA for real-time qPCR analysis. Real-time qPCR analysis utilized the QuantoStudio 7 (Thermo Fisher Scientific, Waltham, MA, USA). The mRNA expression levels were quantified through real-time qPCR with specific primers, and gene expression was normalized to the housekeeping gene GAPDH. Analysis was conducted using the ^2^(−ΔΔCt) method.

### 2.8 RNA-seq analysis

RNA-seq was independently performed on both macrophages and NHDF to analyze gene expression profiles. Total RNA was extracted from macrophages and NHDF utilizing the Maxwell^®^ RSC Instrument (Promega, Madison, WI, USA). The mRNA was then purified using oligo dT beads (NEBNext Poly (A) mRNA Magnet Isolation Module, New England Biolabs, NEB, Ipswich, MA). Complementary DNA (cDNA) library preparation was performed using the NEBNext Ultra II RNA Library Prep Kit (NEB) along with NEBNextplex Oligos for Illumina according to a previously established method (9). Index sequences were incorporated into the fragments through PCR amplification. The cDNA libraries were combined in equal molecular amounts and sequenced using Illumina platforms. For macrophage samples, sequencing was performed on the Illumina NextSeq 500 system (Illumina, San Diego, CA), while NHDF samples were sequenced using the Illumina NovaSeq 6000 platform (Illumina, San Diego, CA). The resulting reads were analyzed using CLC Genomics Workbench software (ver. 8.01, Qiagen, Venlo, The Netherlands). Statistical differences were analyzed by multiple unpaired t-tests using Prism (version 10.0; GraphPad Software, Boston, MA, USA). Gene pathway analysis was performed using Ingenuity Pathway Analysis (Qiagen, Venlo, The Netherlands). The heat map displayed log2(TPM+1) values for each gene from the RNA-seq data created with Prism. A volcano plot was produced with log2(fold change) on the x-axis and -log10(p-value) on the y-axis using Origin Pro 2023 (Northampton, MA). Principal component analysis (PCA) was conducted using JMP Pro 18 (SAS Institute Cary, CA) to validate intragroup variance.

### 2.9 Neutralizing antibody assay

NHDF were plated in 10% FBS-DMEM medium and allowed to grow for 24 hours. Afterward, the medium was switched to serum-free DMEM for another 24 hours of incubation. The cells were then pretreated with or without a neutralizing anti-TLR4 antibody (R&D Systems, AF1478, Minneapolis, MN, USA) in 1% FBS-DMEM for 30 minutes. Next, the medium was replaced with serum-free DMEM containing M1 macrophage-conditioned medium (M1-CM), with or without HA4, or with a control medium lacking M1-CM. After a 6-hour incubation, total RNA was extracted using the Maxwell^®^ RSC Instrument (Promega, Madison, WI, USA), following the manufacturer’s guidelines. The expression levels of IL-6 mRNA were quantified using real-time qPCR with specific primers. Gene expression was normalized to a housekeeping gene and analyzed via the ^2^(−ΔΔCt) method.

### 2.10 Collagen fiber formation assay

NHDF were cultured in plates for 6 days in 10% FBS-DMEM, supplemented with or without conditioned medium and HA4. After this culture period, the cells were fixed in 4% paraformaldehyde and immunostained with an anti-collagen antibody (Sigma-Aldrich, C2456, St. Louis, MO, USA). Fluorescent images were captured using the ImageXpress Micro Confocal High-Content Imaging System (Molecular Devices, San Jose, CA, USA). For each experimental condition, sixteen fields of view were imaged using a 20x objective and the appropriate filter sets for the collagen immunostaining fluorophore. The images were then analyzed using MetaXpress Software (Molecular Devices, San Jose, CA, USA).

### 2.11 Collagenase activity assay

On day 6 of the collagen formation assay, the supernatant obtained from NHDF was utilized to assess collagenase activity via a collagenase activity assay kit (Cosmo Bio Co., Ltd., AK37, Otaru, Japan). The procedure followed the manufacturer’s guidelines outlined in the accompanying manual.

### 2.12 Statistical analysis

Data are expressed as mean ± standard deviation (SD). The pixel intensity of collagen fibers was compared between the M1 and M1+HA4 groups using Student’s *t*-test. A one-way ANOVA was performed using Prism (version 9.0; GraphPad Software, Boston, MA, USA) to assess differences among three or more groups, including the control. Statistical significance was defined as *p* < 0.05.

## 3 Results

### 3.1 Comparison of gene expression in M0 and M1 macrophages and the effects of HA4

M0 macrophages are undifferentiated and can develop into different macrophage types (like M1 or M2) when triggered by stimuli such as bacterial infections or inflammatory responses. Regarding photoaging, studies indicate that UV exposure disrupts the M1/M2 macrophage balance, leading to a higher proportion of M1 macrophages and a lower proportion of M2 macrophages (8). To assess how HA4 influences the differentiation of M0 macrophages into M1 macrophages, several experimental groups were established (1): the M0 group (2), the group treated solely with M1-inducing factors, and (3) the group that received both M1-inducing factors and HA4. RNA-seq analysis was used to compare the gene expression profiles across these groups. PCA indicated that the M1+HA4 group displayed unique PCA components when contrasted with the M0 and M1 groups (**Figure 1B**). Additionally, volcano plot and heatmap analyses demonstrated significant variations in gene expression profiles between the M0 and M1 groups (**Figures 1C, D**).

Network analysis revealed a significant connection between multiple genes and IL-6 expression in the M1 group, while those in the M1+HA4 group exhibited a decrease (**Figures 1E, F**). Furthermore, integrated network analysis indicated that in the M1+HA4 group, the SOCS1 gene was activated (**Figure 1G**).

### 3.2 HA4 modulates M1 macrophage differentiation and reduces marker expression

To enhance understanding of HA4’s impact on M1 differentiation, gene expression analyses were conducted among the M0, M1, M2, and M1+HA4 groups. The differentiation into M0, M1, and M2 macrophages was validated with specific markers (**Supplementary Figures S1B, C**).

**Figure 2:**
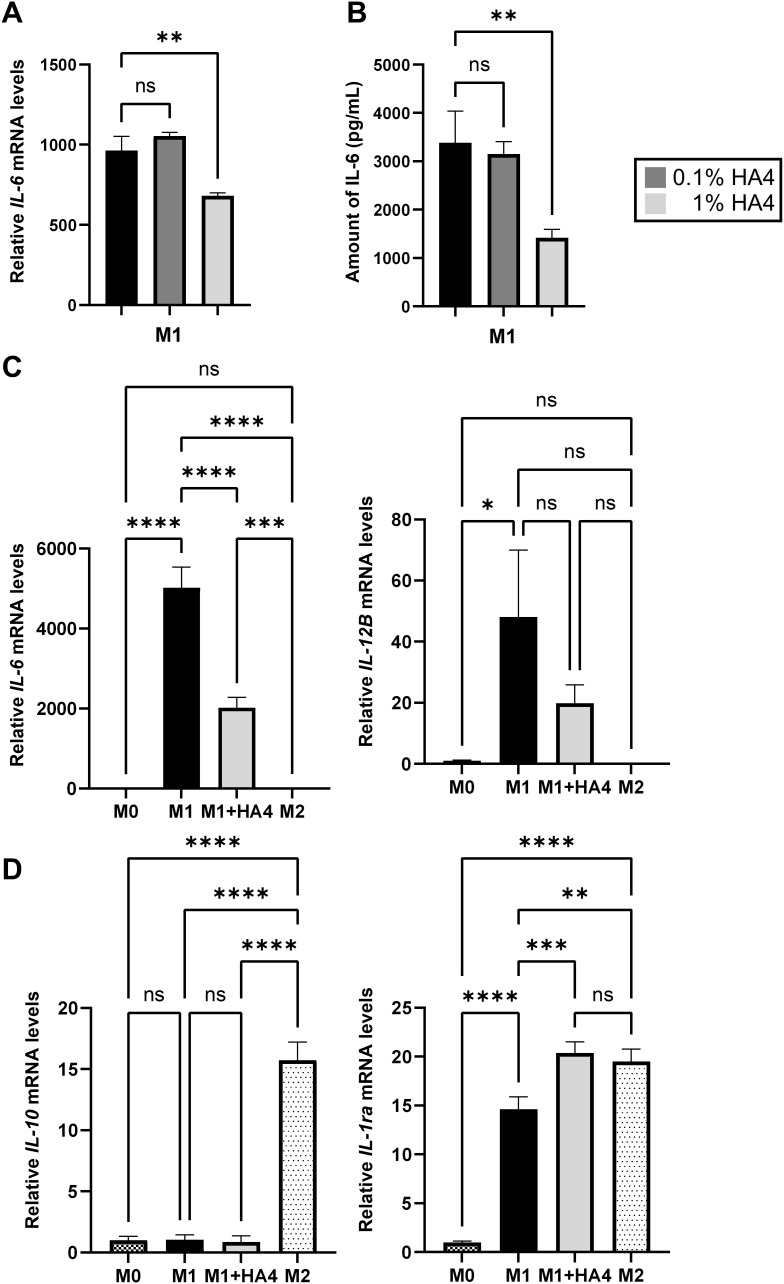
Impact of hyaluronan on macrophage differentiation and cytokine expression profiles. **(A, B)** Analysis of IL-6 expression in HA4-treated M1 macrophages. IL-6 **(A)** gene expression and **(B)** protein levels were assessed in M1 macrophages that were differentiated with 0.1% or 1% HA4. **(C, D)** Comparative assessment of macrophage polarization markers. The mRNA levels of **(C)** M1-related markers (IL-6, IL-12B) and **(D)** M2-related markers (IL-10, IL-1ra) were evaluated in M0, M1, M1+HA4, and M2 macrophages. The bar colors denote different macrophage populations: M0 (dark checkered), M1 (black), M1+HA4 (0.1%) (dark gray), M1+HA4 (1%) (light gray), and M2 (light dotted). Data are shown as mean ± standard deviation (SD) (n = 3). One-way ANOVA was employed for statistical analysis, with significance marked as *p < 0.05, **p < 0.01, ***p < 0.001, ****p < 0.0001. ns, not significant.

To investigate the impact of HA4 on M1 differentiation, we conducted concentration-dependent analyses using qPCR and ELISA. The qPCR analysis showed that IL-6 mRNA expression in the 0.1% HA4 group did not differ significantly from that in the M1 group. In contrast, the 1% HA4 group exhibited a significant reduction in IL-6 mRNA expression (*p* < 0.01, 0.71-fold) (**Figure 2A**). Likewise, IL-12B mRNA expression did not demonstrate significant differences in the 0.1% and 1% HA4 groups compared to the M1 group. However, a trend toward a decrease was observed as the HA4 concentration increased (**Supplementary Figure S1D**). Protein expression analysis through ELISA revealed that IL-6 protein levels in the 0.1% HA4 group did not differ significantly from those in the M1 group; however, there was a significant reduction observed in the 1% HA4 group (*p* < 0.01, 0.42-fold) (**Figure 2B**). In contrast, TNFα protein expression did not show significant differences in the 0.1% HA4 or 1% HA4 groups when compared to the M1 group, although it appeared to decrease as HA4 concentration increased (**Supplementary Figure S1E**).

qPCR analysis revealed that the M1 markers IL-6, IL-12B, TNFα, and IL-8 had significantly higher expression levels in the M1 group compared to the M0 group (*p* < 0.05). However, in the M2 group, their levels were comparable to those in the M0 group (**Figure 2C**, **Supplementary Figure S1F**). Importantly, IL-6 mRNA expression was markedly lower in the M1+HA4 group than in the M1 group (*p* < 0.0001, 0.40-fold) (**Figure 2C**). Conversely, TNFα and IL-8 mRNA expression levels did not differ significantly between the M1 and M1+HA4 groups (**Supplementary Figure S1F**). While IL-12B mRNA levels showed no significant variation between the M1 and M1+HA4 groups, a decreasing trend was noted (**Figure 2C**).

The M2 marker expression was also analyzed. Although HA4 did not significantly influence IL-10 expression, the gene expression of IL-1ra was significantly elevated in the M1+HA4 group compared to the M1 group (*p* < 0.001, 1.39-fold) (**Figure 2D**).

### 3.3 Proinflammatory gene expression induced by M1-CM in NHDF and the role of HA4 in decreasing

**Figure 3:**
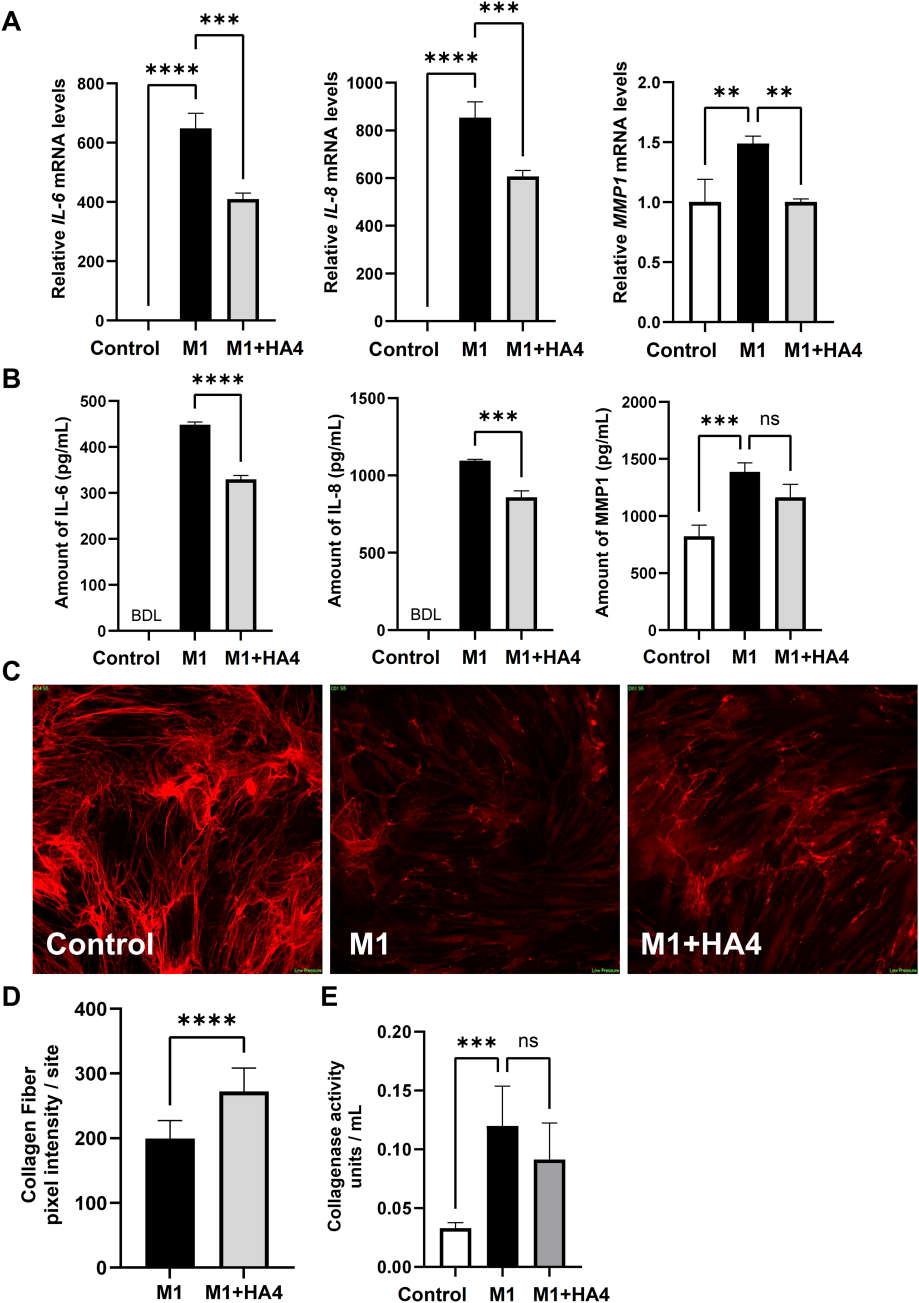
Effects of HA4 on M1 macrophage-induced inflammatory responses and collagen fiber synthesis in NHDF. **(A, B)** mRNA levels, including **(A)** mRNA expression of IL-6, IL-8, and MMP1, and **(B)** protein levels in NHDF exposed to M1 macrophage-conditioned medium (M1-CM) or HA4-treated M1 macrophage-conditioned medium (M1+HA4-CM) (n = 3). NHDF were exposed to the CM diluted 1/64. **(D)** Quantification results (n = 26/27 site) and **(C)** representative images illustrating collagen fiber formation in NHDF subjected to various macrophage-CMs diluted 1/16. **(E)** Collagenase activity in NHDF exposed to either M1-CM or M1+HA4-CM, showing representative data from three independent experiments (n = 6). NHDF were exposed to the CM diluted 1/16. Bar colors indicate the treatment conditions: Control (white), M1 (black), and M1+HA4 (light gray). Data are shown as mean ± standard deviation (SD). Statistical analysis for **(A, B, E)** was performed using one-way ANOVA, whereas **(D)** involved the Student’s *t*-test to compare collagen fiber pixel intensity between M1 and M1+HA4. Significance is indicated as **p < 0.01, ***p < 0.001, ****p < 0.0001. ns, not significant.

Subsequently, we examined how M1 macrophages, known to contribute to skin inflammation during photoaging, affect NHDF through their conditioned medium. Treatment of NHDF with M1-conditioned medium (M1-CM) significantly increased the gene expression levels of the proinflammatory cytokines IL-6 and IL-8, which rose by 648.57-fold and 854.11-fold, respectively, compared to the untreated Control group after 3 hours. The collagen-degrading enzyme MMP1’s expression increased by 1.49-fold (**Figure 3A**).

Furthermore, when the macrophage-conditioned medium, which includes M1-inducing factors and HA4 (designated as M1+HA4), was introduced, the gene expression levels of IL-6, IL-8, and MMP1 significantly decreased, showing reductions of 0.63-fold, 0.71-fold, and 0.67-fold, respectively, when compared to the M1-CM treatment group (**Figure 3A**). Correspondingly, assays for protein quantification indicated that the expression levels of IL-6, IL-8, and MMP1 in the group treated with M1+HA4-conditioned medium (referred to as M1+HA4-CM) were lowered by 0.74-fold, 0.78-fold, and 0.84-fold, respectively, in comparison to the M1-CM-treated group (as shown in **Figure 3B**).

These findings showed that HA4 decreases the expression of proinflammatory cytokines and collagen-degrading enzymes triggered by M1 factors.

### 3.4 M1-CM significantly suppresses collagen fiber formation, and the HA4 addition to M1 macrophage attenuates this suppressive effect

M1-CM’s role in photoaging with inflammation was highlighted by its ability to decrease NHDF’ collagen fiber development. Images in **Figure 3C** illustrate collagen fibers revealed through immunofluorescence staining, taken 6 days after the conditioned medium’s introduction. The introduction of M1-CM notably decreased collagen fiber formation in NHDF (**Figure 3C**). In contrast, the M1 + HA4 group showed a marked increase in collagen fibers compared to the M1-CM group. Quantitative analysis of collagen fiber fluorescence indicated a statistically significant recovery of collagen fiber levels due to HA4 (*p* < 0.0001, **Figure 3D**).

Collagenase activity assays indicated that 6 days after introducing the conditioned medium, the M1-CM group’s collagenase activity was notably higher than that of the Control group (*p* < 0.001, **Figure 3E**).

This experiment was run independently on three occasions to confirm reproducibility. Across all trials, the collagenase activity in the M1+HA4-CM group remained lower than that in the M1-CM group. Nevertheless, no statistical significance was found in individual experiments (*p* > 0.05, n = 3 for each experiment). When the results from these three experiments were aggregated, the collagenase activity in the M1+HA4-CM group showed an average decrease of 18.2%% compared to the M1-CM group (mean ± SD: M1 group, 0.11 ± 0.029; M1 + HA4 group, 0.09 ± 0.025; t-test, p = 0.12; total n = 9).

### 3.5 Gene expression changes in NHDF induced by M1-CM and the effects of HA4

**Figure 4:**
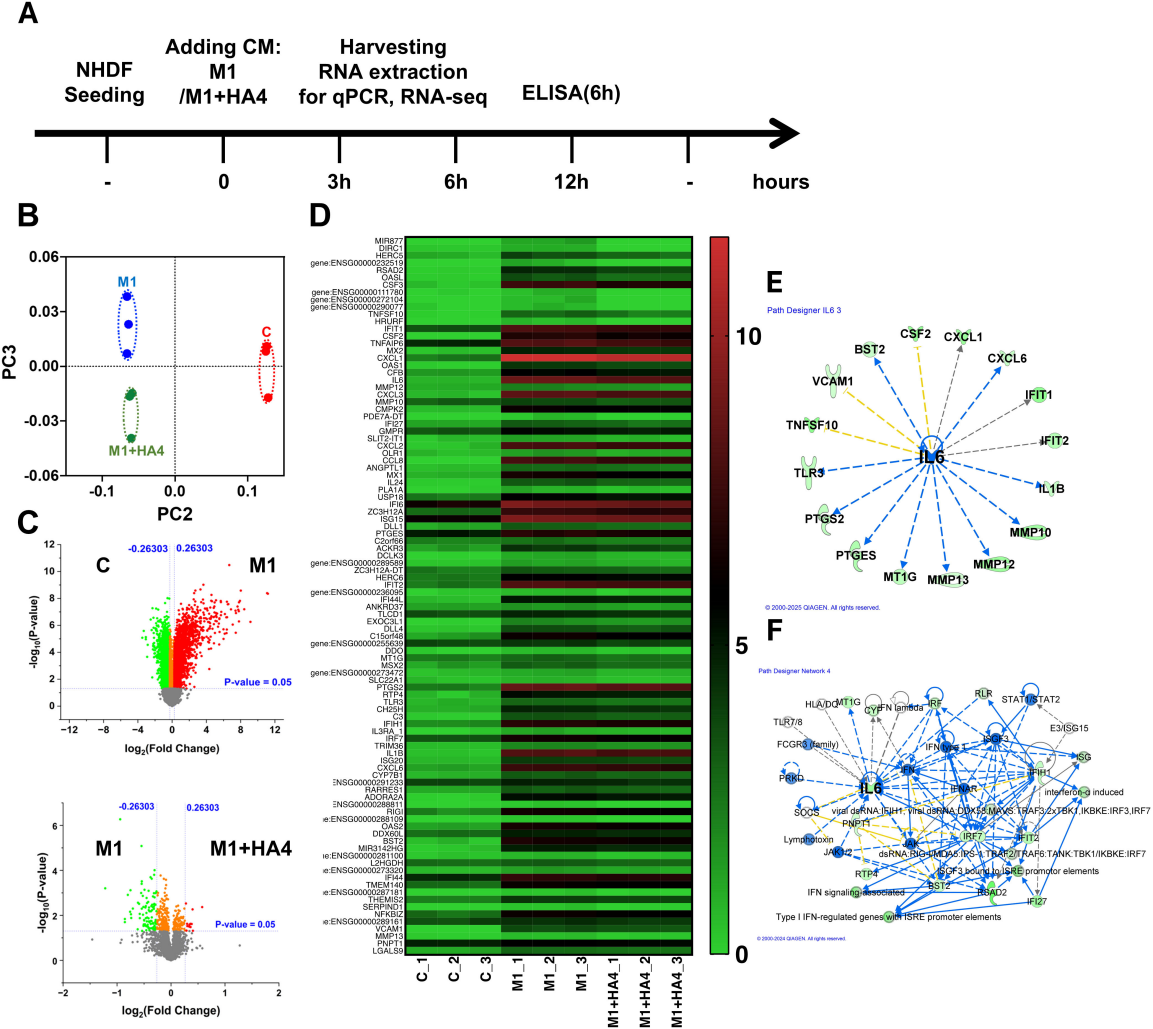
Transcriptomic analysis of NHDF exposed to macrophage-conditioned media. **(A)** A schematic displays the experimental design, outlining the treatment timeline for NHDF with M1-CM or M1+HA4-CM and the time points designated for RNA and protein collection. **(B–F)** RNA-seq analysis was conducted on NHDF 6 hours post-exposure to CM. **(B)** PCA plot representing RNA-seq data, contrasting gene expression profiles of NHDF under various treatment conditions. **(C)** A heatmap illustrating the expression patterns of significantly changed genes across treatment groups. **(D)** Gene regulatory network derived from RNA-seq data analysis, showcasing key interactions and central hubs. **(E, F)** Either **(E)** upstream analysis or **(F)** network analysis underlining IL-6’s role in NHDF treated with M1+HA4. RNA-seq analysis was performed on NHDF (n=3 for each condition), either untreated (Control) or exposed to CM from M0, M1, or other conditions M1+HA4. NHDF were exposed to the CM diluted 1/64.

**Figure 3** illustrates that M1-CM suppresses collagen production while enhancing collagen-degrading enzyme levels in NHDF. These alterations seem linked to the features of photoaged skin. To clarify the mechanisms behind this inflammatory response, we conducted RNA-seq analysis to thoroughly identify the genes in NHDF whose expression changes due to M1-CM and explore the impact of HA4 on these changes in expression (**Figure 4A**).

The RNA-seq analysis results indicated that in the PCA, the M1 and M1+HA4 groups were plotted relatively close to one another compared to the control group. Yet, they exhibited distinct clusters (**Figure 4B**). Volcano plot and heatmap analyses demonstrated that, although the number of differentially expressed genes was lower in the M1 and M1+HA4 groups than in the Control and M1 groups, the introduction of HA4 led to significant changes in the expression of specific genes within the M1 group (**Figures 4C, D**).

It was confirmed that HA4 decreased the expression of genes associated with collagen degradation, specifically IL1B, CXCL1, MMP10, and MMP13, which were induced by M1-CM (**Figure 4D**). Additionally, a network analysis of factors activated in the M1-CM group and downregulated in the M1+HA4-CM group showed a link to IL-6 expression, aligning with the macrophage-related findings shown in **Figure 1** (**Figures 1E, F**, **Figures 4E, F**). IL-6 was recognized as a key factor contributing to the decreased expression of collagen-degrading enzymes like MMP10, MMP12, and MMP13 (**Figure 4E**).

### 3.6 Analysis of gene expression changes in NHDF induced by M1-CM and the effects of HA4

**Figure 5:**
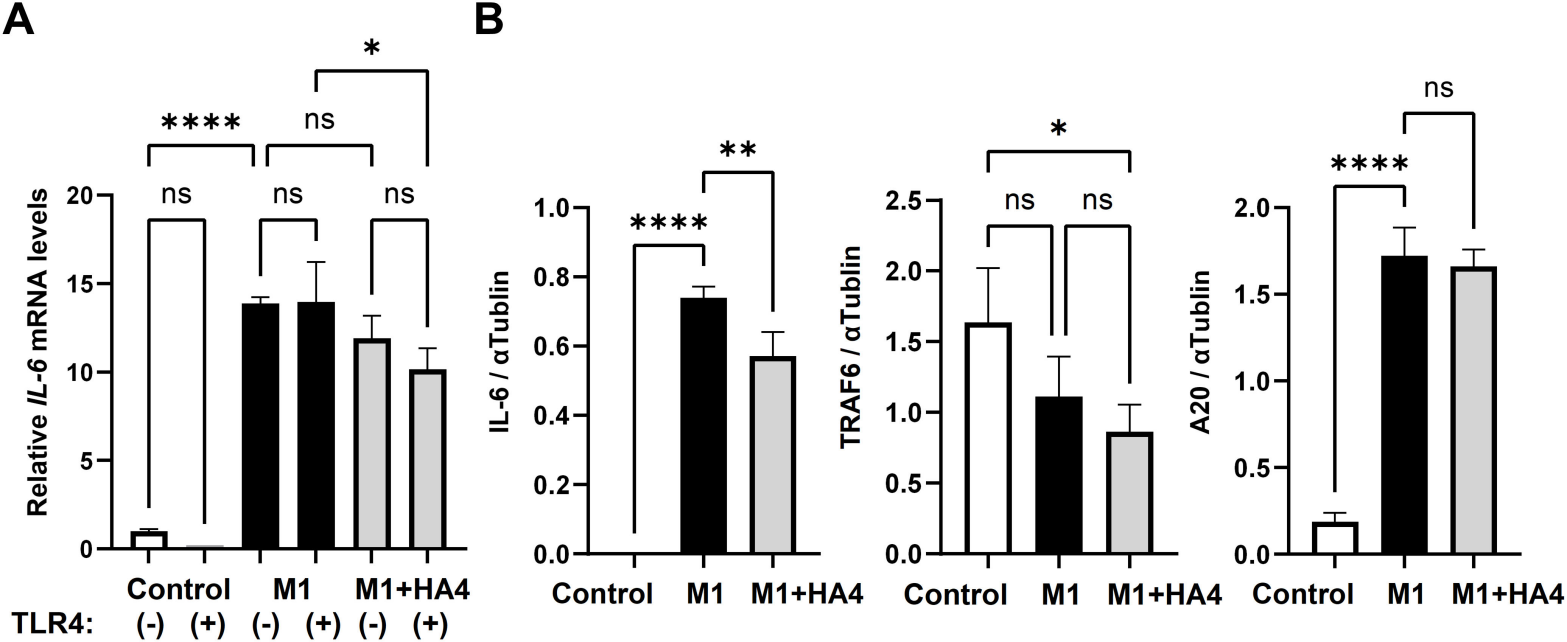
Analysis of TLR4 signaling pathway-related factors and the model diagram. **(A)** Impact of TLR4 neutralizing antibody on IL-6 mRNA expression: NHDF were treated with Control, M1-CM, or M1+HA4-CM, either with or without the anti-TLR4 antibody. A 1/64 dilution of the CM was used to treat NHDF. **(B)** IL-6, TRAF6, and A20 protein levels in NHDF after treatment with Control, M1-CM, or M1+HA4-CM. A 1/16 dilution of the CM was used to treat NHDF. Bar colors indicate different conditions: Control (white), M1 (black), and M1+HA4 (light gray). Data are shown as the mean ± standard deviation (SD) (n = 3). Statistical analysis utilized one-way ANOVA, with significance denoted as *p < 0.05, **p < 0.01, ****p < 0.0001. ns, not significant.

To validate the intracellular signaling pathways associated with the gene expression patterns illustrated in **Figures 2**, **3**, we performed a neutralization assay with TLR4 antibodies. Analysis of IL-6 mRNA expression in NHDF showed that IL-6 levels remained low in the Control group, regardless of the presence of TLR4 antibodies (**Figure 5A**). In the M1-CM-treated group, IL-6 expression was significantly higher than in the Control group. The M1+HA4-CM-treated group exhibited a trend toward decreased IL-6 expression compared to the M1-CM-treated group, though this decline was not statistically significant. Treatment with TLR4 antibodies resulted in reductions in IL-6 levels in the M1-CM and M1+HA4-CM groups; however, these reductions were not statistically significant (**Figure 5A**). These findings imply that the addition of M1 and M1+HA4-CM affects IL-6 expression, yet additional research is required to clarify the role of TLR4 pathway.

We subsequently examined the protein levels of IL-6, TRAF6, and A20 in relation to the TLR4 signaling pathway. TRAF6 and A20 are known to have opposing roles in regulating IL-6 levels. In the Control group, IL-6 protein expression was minimal. In contrast, it significantly increased in the M1-CM-treated group. It decreased markedly in the M1+HA4-treated group (**Figure 5B**). For TRAF6, protein expression was highest in the Control group, then decreased in the M1-CM-treated group, and continued to decline in the M1+HA4-treated group (**Figure 5B**). A20 protein expression was low in the Control group, rose significantly in the M1-CM-treated group, and did not show a statistically significant change compared to the M1+HA4-treated group (**Figure 5B**).

## 4 Discussion

HA is widely found in human tissues, and its moisturizing benefits are especially noted in the skin. Additionally, due to its biocompatibility and biodegradability, HA is commonly utilized as a safe and effective filler in regenerative and aesthetic medicine. Recently, HA has been acknowledged as a potent bioactive molecule that can significantly influence its surrounding environment (1). The type and extent of these effects primarily depend on HA’s molecular weight. Nonetheless, several characteristics of ultralow-molecular-weight HA, particularly HA4, are still unexplored.

### 4.1 Effects of HA4 on M1 macrophage differentiation

This study found that the coadministration of HA4 with stimulatory factors during M1 differentiation significantly reduced both gene and protein expression levels of IL-6 (**Figures 2A–C**). Furthermore, IL-12B expression decreased in a dose-dependent response to HA4 (**Supplementary Figure S1D**). In contrast, other M1 markers like TNFα and IL-8 showed no change in expression (**Supplementary Figure S1E, F**). These findings indicate that HA4 partially suppresses M1 macrophage activation. For M2 markers, HA4 treatment increased IL-1ra expression, while IL-10 levels remained stable (**Figure 2D**). This suggests that HA4 does not fully drive polarization toward the M2 phenotype. However, reports indicate that high-molecular-weight hyaluronan, rather than HA4, promotes M2 polarization. When THP-1 cells are cultured in a specialized three-dimensional environment made of collagen blended with high-molecular-weight HA (HA-COL), HA binds to the receptor CD44, activating the STAT3 gene, which enhances M2 polarization and decreases the expression of M1 markers (TNFa, IL6 IL1B) (10). Macrophage differentiation is dynamic and adapts to alterations in the surrounding microenvironment (11). Our research indicates that HA4 affects macrophage polarization by decreasing M1 activation or facilitating a partial transition from the M1 phenotype to the M2 phenotype. Notably, this transition takes place without entirely driving M2 polarization.

### 4.2 Mechanisms of HA4 action in M1 macrophage differentiation

M1 macrophages express TLR4, and activating its signaling pathway promotes differentiation into the M1 phenotype (11). Our earlier research showed that HA4 competitively binds to TLR4, decreasing damage-associated molecular patterns (DAMPs) released by keratinocytes following UV exposure, suppressing the expression of IL-6 downstream (3). Different cell types participate, yet HA4 might impede the TLR4 signaling pathway linked to IL-6 gene expression during M1 macrophage differentiation. Additionally, RNA-seq analysis, illustrated in **Figure 1G**, indicated that HA4 promotes SOCS1 gene expression during M1 macrophage differentiation. SOCS1 is a negative regulator for multiple Toll-like receptor pathways, including TLR4 (12). To further verify this prediction, we performed real-time qPCR analysis and found that SOCS1 mRNA levels were modestly but significantly elevated in the M1+HA4 group compared to the M1 group (**Supplementary Figure S2**). This supports the idea that HA4 enhances SOCS1 expression. While gene expression analysis alone cannot confirm direct functional protein activity, and additional validation at the protein level is required, our findings imply that HA4 might reduce TLR4 signaling—a crucial pathway for M1 polarization—through the upregulation of SOCS1.

### 4.3 Intracellular signaling pathways of HA4 in NHDF treated with M1-CM

HA interacts with several receptors, including CD44, RHAMM, and TLR4 (1, 2, 13). This study confirmed that M1+HA4-CM decreases IL-6 expression in NHDF (**Figures 3A, B**). The TLR4-dependence of HA4 has been demonstrated in dendritic cells through previous studies utilizing TLR4-knockout mice (14). Based on this, a similar mechanism was hypothesized to exist in macrophages, likewise immune cells. However, experiments using a TLR4-neutralizing antibody in NHDF revealed minimal involvement of TLR4 in this mechanism (**Figure 5A**). Specifically. At the same time, M1-CM increased IL-6 expression in NHDF, but the addition of a TLR4-neutralizing antibody did not change this expression (**Figure 5A**). However, HA4 partially reduced this effect; the changes were not statistically significant (**Figure 5A**).

A detailed examination of TRAF6, a downstream component of TLR4 signaling, showed no meaningful expression difference between NHDF exposed to M1-CM and those exposed to M1+HA4-CM (**Figure 5B**). Additionally, the analysis of A20, an essential factor that decreases TLR4 signaling and TRAF6 function, indicated that TLR4 signaling was inhibited in both scenarios (**Figure 5B**). These findings suggest that the decrease in IL-6 expression by M1+HA4-CM may engage receptors beyond TLR4. It’s plausible that residual HA4 in the M1+HA4 macrophage supernatant is minimal, indicating that the observed effects are likely due to various factors working together within the supernatant. Consequently, to pinpoint the factors influencing IL-6 expression, a thorough evaluation of multiple alternative pathways, including but not limited to TLR4, is essential rather than concentrating on one receptor.

### 4.4 Role of IL-6 in macrophages and NHDF

HA4 was crucial in suppressing IL-6 expression during the differentiation of M1 macrophages (see **Figures 1A, B**) and effectively decreased IL-6 expression induced by M1-CM in NHDF (**Figures 3A, B**). Furthermore, RNA-seq analysis uncovered a network indicating that IL-6 is linked to various factors, underscoring its significance. Importantly, IL-6 was found to be upstream of the decreased expression of ECM-degrading enzymes like MMP7, MMP12, and MMP13 in macrophages (**Figure 1F**) and MMP10, MMP12, and MMP13 in NHDF (**Figure 4E**). This implies that IL-6 not only contributes to inflammation but also plays a role in ECM degradation (15, 16).

IL-6 has a complex role in photoaging, evidenced by various crucial studies. One study revealed that UV exposure raises cytokine levels, including IL-6, in keratinocytes, connecting this rise to activating certain transcription factors, where IL-6 is vital for facilitating skin repair (17). Different opinions exist on how UVA stimulates collagenase activity via IL-6 in fibroblasts. One research study found that UVA increases IL-6 expression, which, through an autocrine mechanism, enhances collagenase activity in fibroblasts. This links the process to UVA-induced skin damage and photoaging. However, the partial inhibition of collagenase activity by IL-6-neutralizing antibodies indicates that other factors may also be involved (18). A study highlighted the essential function of IL-6 released by keratinocytes and fibroblasts in skin sagging caused by UVA exposure (15).

UV radiation produces reactive oxygen species in the skin during photoaging, leading to oxidative stress. IL-6 is a proinflammatory cytokine linked to heightened oxidative stress and inflammation from UV exposure. It contributes significantly to skin damage and aging processes (19).

While this study did not provide a conclusive link between IL-6 expression and the photoaging process in macrophage- NHDF interactions, HA4’s reduced IL-6 expression directed collagen remodeling toward repair and regeneration. These results indicate a promising new therapeutic approach for photoaging.

### 4.5 The beneficial role of HA4 in photoaging

IL-6 and matrix metalloproteinases (MMPs) levels rise in aged or photodamaged skin (20, 21). This study revealed through RNA-seq analysis that in the presence of HA4, the expression levels of MMP-7, MMP-12, and MMP-13 in M1 macrophages were significantly reduced compared to M0 macrophages (**Figure 1D**). MMP-7 and MMP-12 are key enzymes involved in elastin degradation, with MMP-12, known as macrophage elastase, being the most effective elastin-degrading enzyme found in skin (22). MMP-13 is only minimally activated by ultraviolet radiation and exhibits low baseline expression in humans. Nevertheless, it plays a vital role in tissue remodeling largely due to its ability to degrade type II and fibrillar collagen (16). Furthermore, RNA-seq analysis indicated that MMP-10, MMP-12, and MMP-13 expressions were significantly elevated in NHDF treated with M1-CM compared to the Control group. In contrast, the expression of these genes declined in NHDF treated with M1+HA4-CM (**Figure 4D**).

Fibroblasts and keratinocytes exposed to UVA radiation secrete IL-6, which promotes collagen breakdown by increasing MMP-1 expression in fibroblasts. This occurs via autocrine and paracrine mechanisms, leading to skin sagging (15). In this study, inflammatory cytokines such as IL-6, released by M1 macrophages, were found to enhance the expression of MMP-1 in NHDF (**Figures 3A, B**). MMP-1 is the central protease that breaks down type I and III collagen, which are plentiful in human skin. Based on prior research alongside our current results, it is likely that the increased expression of MMP from M1 macrophages and NHDF contributes to the degradation of collagen and elastin fibers in the dermis. This leads to reduced skin elasticity and wrinkles and sagging associated with photoaging. Notably, treatment with M1-CM for 6 days significantly decreased collagen fiber formation in NHDF (**Figure 3C**).

**Figure 6:**
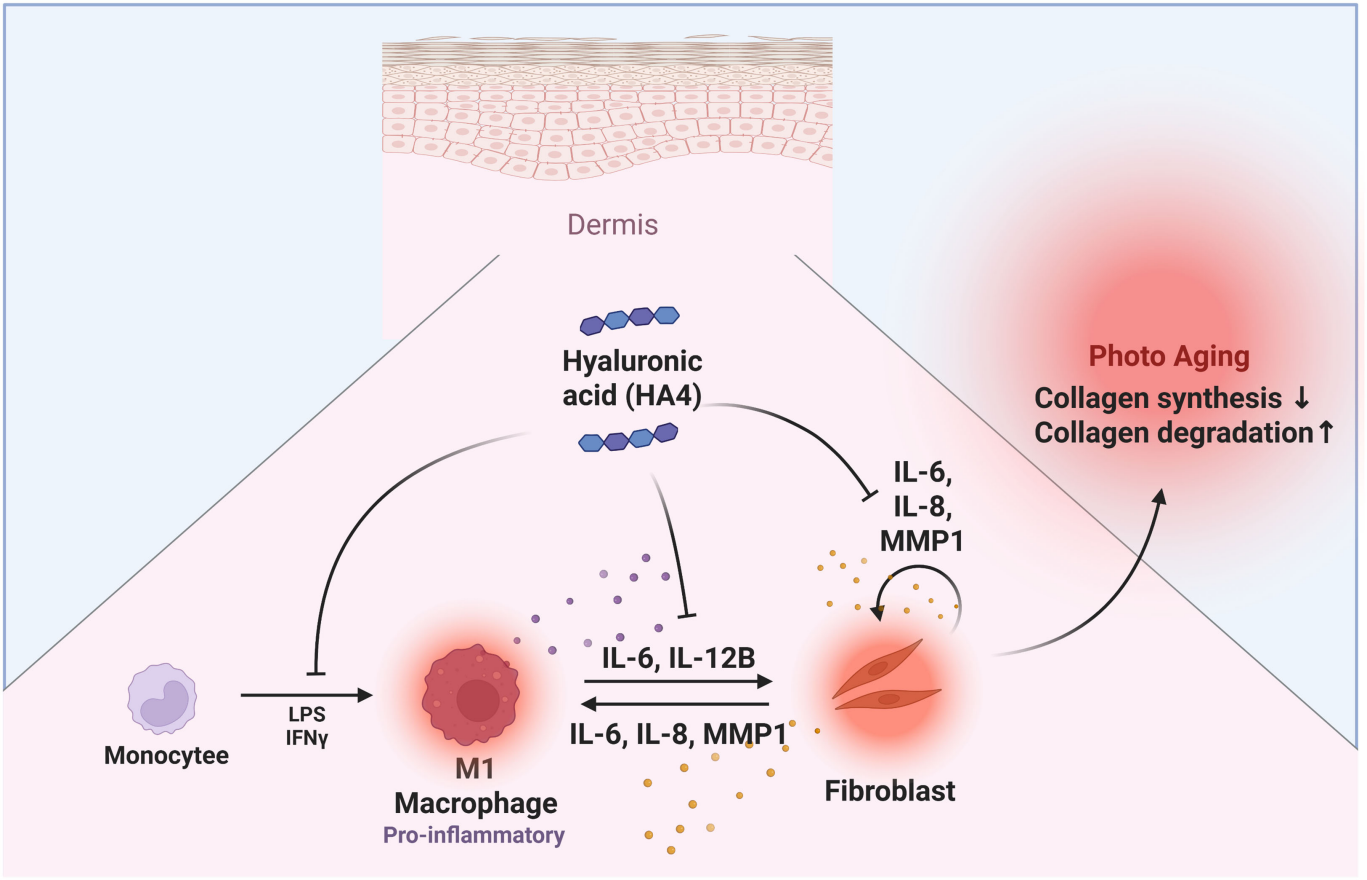
Graphical abstract. Diagram illustrating HA4’s effects on dermal photoaging: A summary of HA4’s role in macrophage- NHDF interactions related to photoaging. HA4 partially mitigates M1 macrophage polarization and the release of inflammatory factors, decreasing collagen breakdown and promoting collagen production in NHDF during photoaging. By reducing these effects, HA4 helps preserve dermal integrity and combat photoaging.

Our research shows that HA4 modulates the inflammatory feedback loop through the paracrine interaction between M1 macrophages and NHDF. Specifically, HA4 partially reduces the differentiation of M1 macrophages and thus limits the release of inflammatory factors from these cells. This reduction indicates that HA4 effectively controls collagen production inhibition and collagen degradation promotion in NHDF, driven by inflammatory factors from M1 macrophages (**Figure 6**). Consequently, these findings establish HA4 as a significant molecule for slowing the photoaging process and alleviating factors that speed up progression. On the other hand, the current results suggest that HA4 may play a role in modulating photoaging by influencing macrophage-driven inflammation and collagen remodeling.

However, this study did not explore the impact of HA4 on other important mechanisms related to photoaging, including oxidative stress and UV-induced DNA damage. Additional research is needed to assess whether HA4 protects against these factors. Investigating these aspects would enhance our understanding of HA4’s anti-photoaging abilities and could expand its therapeutic or cosmetic applications.

### 4.6 Study limitations and future directions

This study has a few limitations. The observed effects were temporary initially, and more research is necessary to assess HA4’s long-term impact on photoaging suppression. Additionally, the effect of M1-CM on epidermal cells is unexplored, presenting an interesting direction for future studies. By enhancing our knowledge of how HA4 influences keratinocytes, melanocytes, and fibroblasts, we can better understand the mechanisms through which HA4 mitigates photoaging, ultimately leading to a more thorough comprehension of its therapeutic potential. Moreover, it is important to recognize that various factors—such as ultraviolet radiation, oxidative stress, chronic inflammation, and the breakdown of the extracellular matrix—affect skin aging. Thus, it is improbable that HA4’s action alone can clarify these intricate mechanisms entirely. Future research should incorporate HA4’s role with other crucial pathways, like oxidative damage and mitochondrial dysfunction, to gain a deeper insight into the processes that drive skin aging.

A significant limitation of this study is that it lacks *in vivo* or clinical validation. The choice to forgo animal experiments was made for ethical reasons and by institutional policies that promote alternatives to animal testing. Although we recognize that clinical evidence is crucial for translational applications, such studies were not feasible for this research due to limited resources. Nevertheless, the *in vitro* collagen production assay used here is a well-accepted surrogate for early-stage efficacy evaluation and offers valuable insights into the biological impacts of the HA4-treated supernatant. Future research should focus on validating these results in suitable animal models and eventually in human clinical trials to establish the therapeutic potential observed in this study.

## Data Availability

The data presented in the study are deposited in the GEO repository, accession numbers GSE298073 (macrophages) and GSE298076 (NHDFs).

## References

[1] Muto J Sayama K Gallo RL Kimata K. Emerging evidence for the essential role of hyaluronan in cutaneous biology. J Dermatol Sci. (2019) 94:190–5. doi: 10.1016/j.jdermsci.2019.01.009 30935779

[2] Abatangelo G Vindigni V Avruscio G Pandis L Brun P. Hyaluronic acid: Redefining its role. Cells. (2020) 9:1743. doi: 10.3390/cells9071743 32708202 PMC7409253

[3] Hu L Nomura S Sato Y Takagi K Ishii T Honma Y. Anti-inflammatory effects of differential molecular weight Hyaluronic acids on UVB-induced calprotectin-mediated keratinocyte inflammation. J Dermatol Sci. (2009) 107:24–31. doi: 10.1016/j.jdermsci.2022.06.001 35717315

[4] Frenkel JS. The role of hyaluronan in wound healing. Int Wound J. (2012) 11:159–63. doi: 10.1111/j.1742-481X.2012.01057.x PMC7950635 22891615

[5] Campo GM Avenoso A Campo S D’Ascola A Nastasi G Calatroni A. Small hyaluronan oligosaccharides induce inflammation by engaging both toll-like-4 and CD44 receptors in human chondrocytes. Biochem Pharmacol. (2010) 80:480–90. doi: 10.1016/j.bcp.2010.04.024 20435021

[6] Termeer CC Hennies J Voith U Ahrens T Weiss JM Prehm P. Oligosaccharides of hyaluronan are potent activators of dendritic cells. J Immunol. (2000) 165:1863–70. doi: 10.4049/jimmunol.165.4.1863 10925265

[7] Horiba S Kawamoto M Tobita R Kami R Ogura Y Hosoi J. M1/M2 Macrophage skewing is related to reduction in types I, V, and VI collagens with aging in sun-exposed human skin. JID Innov. (2023) 3:100222. doi: 10.1016/j.xjidi.2023.100222 37789949 PMC10542643

[8] Horiba S Kami R Tsutsui T Hosoi J. IL-34 downregulation–associated M1/M2 macrophage imbalance is related to inflammaging in sun-exposed human skin. JID Innov. (2022) 2:100112. doi: 10.1016/j.xjidi.2022.100112 35521044 PMC9062483

[9] Kohno M Kobayashi S Yamamoto T Yoshitomi R Kajii T Fujii S. Enhancing calmodulin binding to cardiac ryanodine receptor completely inhibits pressure-overload induced hypertrophic signaling. Commun Biol. (2020) 3:714. doi: 10.1038/s42003-020-01443-w 33244105 PMC7691336

[10] Kim H Cha J Jang M Kim P. Hyaluronic acid-based extracellular matrix triggers spontaneous M2-like polarity of monocyte/macrophage. Biomater Sci. (2019) 7:2264–71. doi: 10.1039/C9BM00155G 30849138

[11] Wang N Liang H Zen K. Molecular mechanisms that influence the macrophage m1-m2 polarization balance. Front Immunol. (2014) 5:614. doi: 10.3389/fimmu.2014.00614 25506346 PMC4246889

[12] Fujimoto M Naka T. SOCS1, a negative regulator of cytokine signals and TLR responses, in human liver diseases. Gastroenterol Res Pract. (2010) 2010:470468. doi: 10.1155/2010/470468 20862390 PMC2939392

[13] Muto J Morioka Y Yamasaki K Kim M Garcia A Carlin AF. Hyaluronan digestion controls DC migration from the skin. J Clin Invest. (2014) 124:1309–19. doi: 10.1172/JCI67947 PMC3934161 24487587

[14] Papakonstantinou E Roth M Karakiulakis G. Hyaluronic acid: A key molecule in skin aging. Dermatoendocrinol. (2012) 4:253–8. doi: 10.4161/derm.21923 PMC3583886 23467280

[15] Imokawa G Nakajima H Ishida K. Biological mechanisms underlying the ultraviolet radiation-induced formation of skin wrinkling and sagging II: Over-expression of neprilysin plays an essential role. Int J Mol Sci. (2015) 16:7776–95. doi: 10.3390/ijms16047776 PMC4425049 25856676

[16] Feng C Chen X Yin X Jiang Y Zhao C. Matrix metalloproteinases on skin photoaging. J Cosmet Dermatol. (2024) 23:3847–62. doi: 10.1111/jocd.v23.12 PMC11626319 39230065

[17] Salminen A Kaarniranta K Kauppinen A. Photoaging: UV radiation-induced inflammation and immunosuppression accelerate the aging process in the skin. Inflammation Res. (2022) 71:817–31. doi: 10.1007/s00011-022-01598-8 PMC9307547 35748903

[18] Wlaschek M Bolsen K Herrmann G Schwarz A Wilmroth F Heinrich PC. UVA-induced autocrine stimulation of fibroblast-derived-collagenase by IL-6: A possible mechanism in dermal photodamage? J Invest Dermatol. (1993) 101:164–8. doi: 10.1111/1523-1747.ep12363644 7688402

[19] Calvo MJ Navarro C Durán P Galan-Freyle NJ Parra Hernández LA Pacheco-Londoño LC. Antioxidants in photoaging: From molecular insights to clinical applications. Int J Mol Sci. (2024) 25:2403. doi: 10.3390/ijms25042403 38397077 PMC10889126

[20] Quan T Little E Quan H Qin Z Voorhees JJ Fisher GJ. Elevated matrix metalloproteinases and collagen fragmentation in photodamaged human skin: Impact of altered extracellular matrix microenvironment on dermal fibroblast function. J Invest Dermatol. (2013) 133:1362–6. doi: 10.1038/jid.2012.509 PMC3637921 23466932

[21] Gather L Nath N Falckenhayn C Oterino-Sogo S Bosch T Wenck H. Macrophages are polarized toward an inflammatory phenotype by their aged microenvironment in the human skin. J Invest Dermatol. (2022) 142:3136–45. doi: 10.1016/j.jid.2022.06.023 35850208

[22] Van Doren SR. Matrix metalloproteinase interactions with collagen and elastin. Matrix Biol. (2015) 44-46:224–31. doi: 10.1016/j.matbio.2015.01.005 PMC4466143 25599938

