# Supplementary material for: Targeting inflammatory macrophages with hyaluronan tetrasaccharide: effects on fibroblast collagen degradation and synthesis
Source: Front Immunol. 2025 Jun 5;16:1592751. doi: 10.3389/fimmu.2025.1592751 (PMC12176868; doi:10.3389/fimmu.2025.1592751)
Supplement: Supplementary file 1 [file Table1.docx]

Supplementary Material


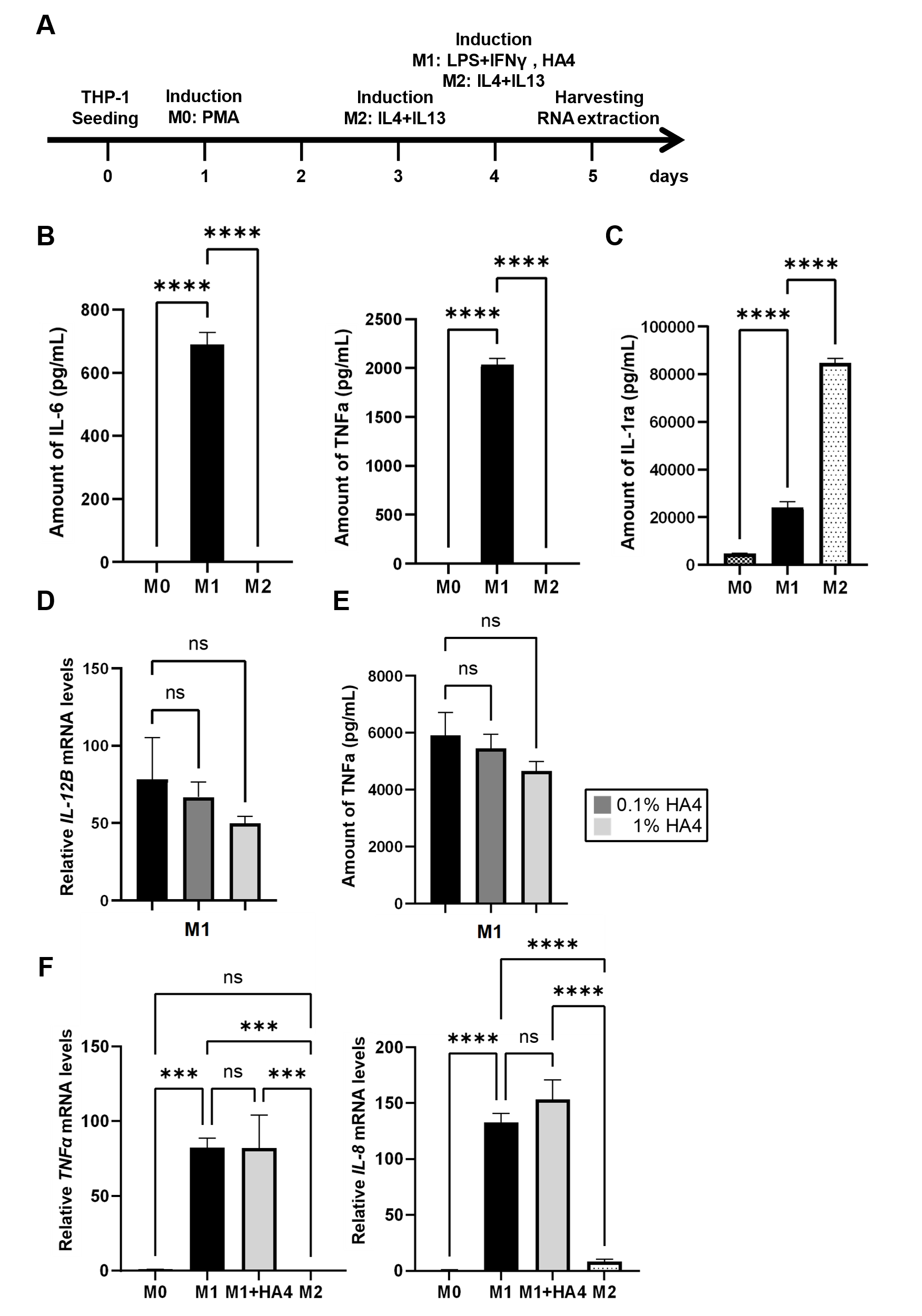


**Supplementary Figure S1. Impact of hyaluronan on macrophage differentiation and cytokine expression profiles**

(A) this schematic illustrates the experimental design for differentiating macrophages from M0 to M1/M2 phenotypes with hyaluronan (HA) treatment.

(B-C) The protein expression levels of (B) M1 markers (IL-6 and TNFα) and (C) the M2 marker (IL-1ra) in the differentiated macrophages are shown.

(D-E) The expression levels of (D) IL-12B mRNA and (E) TNFα protein in M1 macrophages were differentiated with either 0.1% or 1% HA4.

(F) The mRNA expression of M1 markers (TNFα and IL-8) across M0, M1, M1+HA4, and M2 macrophages is represented. The colors of the bars indicate different macrophage populations: M0 (dark checkered), M1 (black), M1+HA4 (0.1%) (dark gray), M1+HA4 (1%) (light gray), and M2 (light dotted). Data is presented as mean ± standard deviation (SD) (n = 3). A one-way ANOVA was conducted for statistical analysis, with significance indicated as *p < 0.05, **p < 0.01, ***p < 0.001, ****p < 0.0001.
