# Supplementary material for: Targeting inflammatory macrophages with hyaluronan tetrasaccharide: effects on fibroblast collagen degradation and synthesis
Source: Front Immunol. 2025 Jun 5;16:1592751. doi: 10.3389/fimmu.2025.1592751 (PMC12176868; doi:10.3389/fimmu.2025.1592751)
Supplement: Supplementary file 2 [file Table2.docx]

Supplementary Material

**Supplementary Figure S2. Impact of hyaluronan on macrophage differentiation and SOCS1 gene expression profile**

(A) The mRNA expression of SOCS1 across M0, M1, M1+HA4, and M2 macrophages is represented. The colors of the bars indicate different macrophage populations: M0 (dark checkered), M1 (black), M1+HA4 (1%) (light gray), and M2 (light dotted). Data is presented as mean ± standard deviation (SD) (n = 3). A one-way ANOVA was conducted for statistical analysis, with significance indicated as ****p* < 0.001, *****p* < 0.0001.
